# A rare case of bladder paraganglioma treated successfully with robotic partial cystectomy

**DOI:** 10.1530/EO-24-0044

**Published:** 2025-01-06

**Authors:** Kalyan M Shekhda, Jessal M Palan, Christo B Albor, Simon Wan, Teng-Teng Chung

**Affiliations:** ^1^ Department of Diabetes and Endocrinology, University College London Hospital NHS Foundation Trust, London, UK; ^2^ Department of Diabetes and Endocrinology, Whittington Hospital NHS Foundation Trust, London, UK; ^3^ Department of Diabetes and Endocrinology, Kings College Hospital NHS Foundation Trust, London, UK; ^4^ Department of Radiology and Nuclear Medicine, University College London Hospital NHS Foundation Trust, London, UK

**Keywords:** bladder paraganglioma, partial cystectomy, Ga-DOTATATE scan, robotic partial cystectomy

## Abstract

Bladder paragangliomas are rare extra-adrenal urological tumors that account for around 0.05% of bladder cancers. Their diagnosis is often delayed because of the rarity of these tumors. There is a risk of an intraoperative hypertensive crisis if not diagnosed or identified before surgical removal. We describe a case of a 36-year-old lady presented with a 10-year history of post-micturition palpitations and headaches. Her biochemical workup showed raised urinary normetanephrine levels and imaging showed a ^123^I MIBG-avid bladder mass compatible with bladder paraganglioma, although interestingly almost no tracer was picked up in ^68^Ga DOTATATE imaging. She was started on phenoxybenzamine to control her blood pressure prior to surgery. She underwent a successful robotic partial cystectomy with no complications. After surgery, she remained symptom-free. Bladder paragangliomas are rare neuroendocrine tumors of the bladder, which need to be diagnosed and managed effectively to avoid intraoperative and long-term complications.

**Learning points:**

- It is important for patients with a bladder lesion to have a comprehensive differential assessment and biochemical and radiological investigations including functional imaging.
- Multiple imaging modalities along with what is available are useful in the assessment of bladder paraganglioma.
- The key role of the multidisciplinary team is to plan treatment in the perioperative period for minimizing risk, especially in situations where optimal management is actively debated.

## Background

Paragangliomas are rare neuroendocrine neoplasms that originate from extra-adrenal paraganglia of the parasympathetic and sympathetic nervous systems (Asa *et al.* 2018). The head and neck are common sites of origin. Abdominal sites include peri-adrenal, para-aortic and inter-aortocaval areas, and rarely, they may originate from the urinary bladder (Lawrence *et al.* 2004, Nölting *et al.* 2022, Pang *et al.* 2024s). Paragangliomas of the urinary bladder (PUB) are extremely rare, originating from the Organ of Zuckerkandl – a neural crest derivative within the bladder wall accounting for 0.05% of all bladder cancers and 1% of all phaeochromocytoma and paragangliomas (PPGL) (Asa *et al.* 2018, Pang *et al.* 2024, Zare *et al.* 2024). Because of their rarity and nonspecific symptoms, approximately 50% of patients with PUB are diagnosed only after surgical resection based on histology (Li *et al.* 2022). We present the case of a 36-year-old lady with no other past medical history of note who presented with symptoms post-micturition of dizziness and flushing. She was also found to be hypertensive following micturition. Subsequent investigations confirmed PUB.

## Case presentation

A 36-year-old female of mixed Southeast Asian and South American background was referred to the endocrine clinic for episodic headache and hypertension while micturating. She had a 10-year history of post-micturition signs and symptoms including a rise in home-monitored blood pressure, palpitations, shortness of breath and paresthesia (described by the patient as ‘like she was running for a bus’). These symptoms were associated with thunderclap headache at onset and during micturition. Each episode would spontaneously resolve after 1–2 min. Ambulatory and regular blood pressure monitoring noted spikes in blood pressure up to 169/110 mmHg. She did not have any past medical history of note. There was no family history of pheochromocytoma or paraganglioma. She smoked occasionally, rarely drank alcohol and had two children under four years, one having been treated for acute lymphoblastic leukemia. When reviewed in the endocrine clinic, her pulse was 88 beats per minute and blood pressure 130/90 mmHg, with no other abnormalities on clinical examination.

## Investigations

Her initial biochemical investigations showed raised urinary and plasma normetanephrines, with normal plasma 3-methoxy tyramine, and plasma and urinary metanephrines (Table 1). Given the abovementioned history, after discussion with the urology team, she underwent flexible cystoscopy, which revealed a suburothelial bladder mass.

**Table 1:** Biochemical investigations before and after surgery.

Date	24/08/2023 (initial)	08/09/2023 (presurgery)	23/11/2023 (postsurgery)
Hb (NRR: 115–155 g/L)	119		
Sodium (NRR: 135–145 mmol/L)	139	139	139
Potassium (NRR: 3.5–5.1 mmol/L	4.1	4.3	4.1
Creatinine (NRR: 45–84 mmol/L)	71	73	82
Serum-adjusted calcium (NRR: 2.2–2.6 mmol/L)	2.38	2.33	2.44
Serum parathyroid hormone (NRR: 1.6–6.9 pmol/L)	4		
Serum TSH (NRR: 0.27–4.2 mIU/L)	1.1		
Serum-free T4 (NRR: 12–22 pmol/L)	13.3		
Serum prolactin (NRR: 102–496 mIU/L)	90		
Alanine transaminase (NRR: 10–35 IU/L)	11	12	
25-Hydroxyvitamin D (NRR: 25–120 nmol/L)		54	
Urine volume (mLs)	1,194		
Urinary normetadrenaline (NRR: 0–3.31 mmol/day)	14.85		
Urinary metadrenaline (NRR: 0–1.21 mmol/day)	1.19		
Plasma normetanephrines (NRR: 120–1180 pmol/L)	9,471	7,563	564
Plasma metanephrines (NRR: 80–510 pmol/L)	363	254	193
Plasma 3-methoxytyramine (NRR: 0–180 pmol/L)	<180	<180	<180

Abbreviations: NRR, normal reference range; Hb, hemoglobin; TSH, thyroid-stimulating hormone.

**Figure 1:**
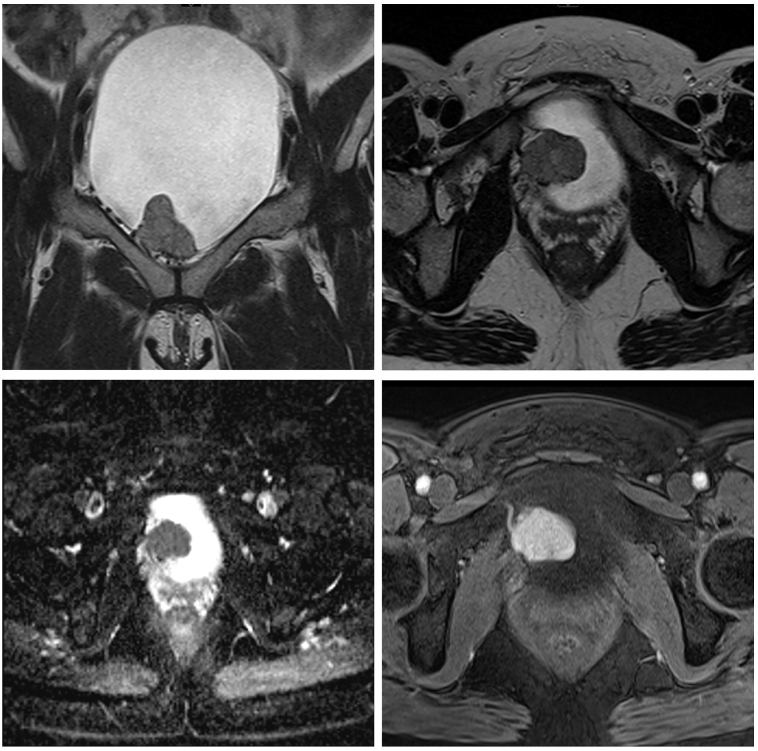
Clockwise from top right: coronal and axial T2 weighted images, T1 fat suppressed post-contrast image and apparent diffusion coefficient image; this shows the soft tissue tumor arising from right inferior aspect of the bladder with a feature of high vascularity and restricted diffusion.

**Figure 2:**
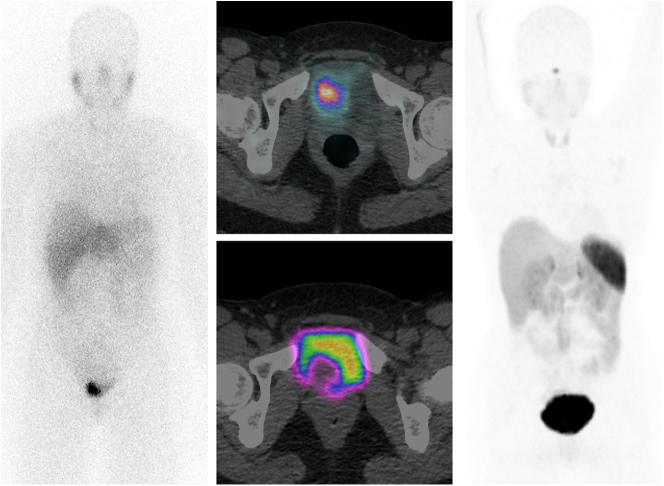
^123^I MIBG whole-body planar scintigraphy and fused axial image at the level of the bladder mass (left and top middle); ^68^Ga DOTATATE whole-body PET/CT three-dimensional maximum intensity projection image and fused axial image at the level of the bladder mass (right and bottom middle). These show the bladder tumor to be showing intense uptake of ^123^I MIBG but little uptake of ^68^Ga DOTATATE. Abbreviations: ^123^I MIBG, iodine-123-metaiodobenzylguanidine; ^68^Ga DOTATATE PET/CT, tetraazacyclododecane tetraacetic acid–DPhe1-Tyr3-octreotate positron emitting tomography.

Initial imaging was via ^123^I MIBG (iodine-123-metaiodobenzylguanidine) followed by MRI (magnetic resonance imaging) and SPECT-CT (single photon emission computed tomography) and ^68^Gallium DOTATATE (gallium-68-tetraazacyclododecane tetraacetic acid–octre-otate) PET/CT (positron emission tomography overlayed with computed tomography). The MRI of the urinary bladder revealed the 33 mm lobulated lesion (Fig. 1). This was arising from the right lateral bladder wall at the base, extending towards the bladder neck, demonstrating marked restricted diffusion and avid enhancement, representing a bladder tumor. Adjacent serpiginous signal voids reflected neo-angiogenesis. The disruption of the low-T2-signal muscularis layer inferiorly reflected muscle involvement with no obvious extravesical extension. The urinary bladder was otherwise thin-walled, with no other focal lesion. ^123^I MIBG SPECT-CT of the abdomen and pelvis showed an intensely avid bladder mass measuring up to 4.3 cm in size, suggestive of a bladder paraganglioma. ^68^Ga DOTATATE PET/CT did not reveal any avid lesions around the bladder or anywhere else in the body (Fig. 2).

## Treatment

After input from the multidisciplinary team (MDT) from endocrinology, endocrine surgery, radiology and urology, she was started on phenoxybenzamine 10 mg twice a day (BD) for alpha-blockade, which was later titrated up to 10 mg four times a day (QDS) and then 20 mg QDS thereafter. Her symptoms improved considerably on starting medication. After further MDT discussion, robotic partial cystectomy and removal of the lesion were planned. Her peri-operative optimization with alpha-blocking agents and close hemodynamic monitoring was essential in this high-risk procedure. The general anesthetics was managed by our highly experienced pheochromocytoma anesthetist who was well rehearsed with this type of tumor. She underwent surgery with no peri- or postoperative complications.

## Outcome and follow-up

Histology from samples showed a well-circumscribed 35 mm nodular piece of tumor with a solid cut surface and focal hemorrhage. The lamina propria and muscularis propria showed a well-circumscribed tumor composed of nests of cells with moderately pleomorphic vesicular nuclei, prominent nucleoli and granular cytoplasm and an intervening vascular network. The cells also showed focal spindling. No necrosis or convincing lymphovascular invasion was seen. Mitoses were scarce (up to 1 per 10 HPF). Local excision appeared complete by 1 mm at the nearest circumferential inked surgical limit. A panel of immunocytochemistry showed the cells were positive for CD56, synaptophysin and chromogranin and negative for calretinin, inhibin and melan A. S100 was positive in some of the cells and scattered sustentacular cells. The proliferation fraction with MIB1 was about 1%. The appearances and immunoprofile were consistent with paraganglioma of the bladder, stage pT2, narrowly excised. Genetic testing for a panel of genes known to cause inherited phaeochromocytoma and paraganglioma (*DLST, FH, MAX, MDH2, MEN1, SDHA, SDHAF2, SDHB, SDHC, SDHD, SLC25A11, TMEM127* and *VHL* genes and exons *5, 7, 8, 10, 11, 13, 14, 15* and *16* of the *RET* gene) did not identify any pathogenic variant.

Her postoperative imaging (abdomen and pelvis MRI) showed complete resection of bladder tumor with no evidence of local recurrence. She was followed up in an endocrine and urology clinic every 6 months. Her postoperative biochemical investigations were normal including normal plasma metanephrine levels (Table 1).

## Discussion

PUBs were first described by Zimmerman and coworkers in 1953 as a very rare type of bladder tumor (Zimmerman *et al.* 1953). Although the age of onset ranges from 7 to 81 years, most patients are diagnosed between the ages of 25 and 65 years (Li *et al.* 2022), with a higher incidence in women than in men (Male *et al.* 2019). In a recent 2022 systematic review of 177 papers (194 cases), only about half of the patients with PUB were found to have symptoms related to episodic catecholamine secretion triggered by urination, including symptomatic hypertension, dizziness and sweating (Li *et al.* 2022). Our patient did have such symptoms, notably episodic hypertension and sweating – indeed it was the intermittent blood pressure spikes in her ambulatory blood pressure monitoring that prompted further investigation for paraganglioma. It is worth noting that around half of the patients with PUB present with nonspecific symptoms such as hematuria and are diagnosed with PUB on tumor histology, only after surgical intervention. The 2022 systematic review has shown this compellingly (Li *et al.* 2022). The summary of a collection of cases (excluding those which were included in the systematic review (Li *et al.* 2022)) from across the globe (China (Hu *et al.* 2022), India (Shrivastava *et al.* 2020, Misgar *et al.* 2022), Jordan (Alkhatatbeh *et al.* 2020), Iran (Zare *et al.* 2024), Canada (Malhotra *et al.* 2018) and Colombia (Pérez Barón *et al.* 2024)) provides further examples of the various presentations leading to investigations with low sensitivity for PUB identification and thus late diagnoses (Table 2).

**Table 2:** Selected case reports.

Reference	Place	Age	Presentation (significant past medical history)	Order of investigations before surgery (* = diagnostic)	Surgery	Pre-surgical alpha-blockade? (BP/arrythmogenic complications)	Investigation revealing diagnosis	Outcome
Hu *et al.* (2022)	China	52 F	Palpitations, exhaustion, emotional agitation and urination (atrial fibrillation)	(1) US (2) CT (3) Urine metanephrines (normal)	Partial cystectomy	Yes (labile BP intraoperatively)	Unclear. Histology not reported	Resolution of symptoms. No recurrence after 6 months
Misgar *et al.* (2022)	India	52 F	Palpitations, headache, dizziness, pallor and symptoms associated with micturition	(1) US (2) *Urine metanephrines (3) MRI	Subtotal cystectomy	Yes (single episode of atrial flutter as inpatient prior to surgery)	Urine metanephrines	Resolution of symptoms. No recurrence
Shrivastava *et al.* (2020)	India	55 F	Dysuria and abdo pain	(1) US (2) CT (3) *Cystoscopy & biopsy	Partial cystectomy	Yes (no complications)	Histology of biopsy from cystoscopy	Not specified
Alkhatatbeh *et al.* (2020)	Jordan	62 M	Obstructive urinary symptoms, poor stream, hesitancy and straining	(1) US (2) CT (3) MRI (4) Cystoscopy	(1) TURBT (histology mistakenly showed transitional cell carcinoma) (2) TURBT (histology then revealed paraganglioma) (3) TURBT	Yes, but only for third operation (labile BP during first and second operations, but no complications for third operation)	Histology of tissue from second operation	Resolution of symptoms. No recurrence
Zare *et al.* (2024)	Iran	37 M	Postmicturition palpitation, headache and sweating	(1) US (2) CT (3) Serum & urine catecholamine (normal) (4) Cystoscopy	Surgery (not specified)	Yes (not specified whether there were complications)	Histology after surgery	Not specified
Malhotra *et al*. (2018)	Canada	12 M	Gross hematuria and vomiting acutely 1-year history position induced headaches, polyuria, polydipsia, nocturia and lethargy	(1) US (2) *Cystoscopy + biopsy (3) 18F-FDG PET (uptake at tumor site)	Partial cystectomy	Yes (not specified whether there were complications)	Histology of biopsy from cystoscopy (noted labile BP during cystoscopy) SDHB mutation identified postsurgically	Recurrence 3 years postresection, bladder wall and inguinal canal Had been monitored with urine metanephrines. Further surgery and continued monitoring
Pérez Barón *et al.* (2024)	Colombia	27 M	Hematuria and vertigo (hypertension)	(1) *Urine metanephrines (2) CT	Laparoscopic partial cystectomy and robotic-assisted lymphadenectomy	Not specified whether alpha-blockade used (not specified whether there were complications)	Urine metanephrines	Not specified
Matsuzawa *et al*. (2022)	Japan	64 M	Weight loss with (hypertension and arrythmia)	(1) CT (2) MRI (3) Cystoscopy (4) 18F-FDG PET/CT (4) EUS-FNA.	Laparotomy: total cystectomy and anterior resection of the rectum	No (labile BP intra-operatively)	On histology postsurgically Biopsy from EUS-FNA histology suggested GIST tumor	No tumor recurrence at 7-month follow-up

Abbreviations: BP, blood pressure; TURBT, trans-urethral resection of bladder tumor; US, ultrasound; CT, computed tomography; MRI, magnetic resonance imaging; ^18^F-FDG PET, fludeoxyglucose 18 positron emission tomography; EUS-FNA, endoscopic ultrasound-guided fine needle aspiration.

PUBs can be diagnosed, localized and metastases screened for, based on various imaging modalities. Ultrasound scans of the urinary system can show the hypervascular nature of these tumors mostly located along the anterior or posterior bladder wall (Malhotra *et al.* 2018, Zare *et al.* 2024). Contrast-enhanced computed tomography has a sensitivity of 91% and can show hyperdense rounded, homogeneous lesions alongside enhancement in the arterial phase with perilesional neovascularization. Although necrosis is rare, about 10% of the cases have calcification (Pérez Barón *et al.* 2024). MRI is more sensitive than CT and is an excellent imaging modality for tumor localization within bladder wall layers. They typically appear hyperintense on T2 and T1 weighted images, compared with the muscularis propria. Diffusion restriction can be observed, and larger tumors may exhibit a ‘salt and pepper’ appearance (Qin *et al.* 2020, Matsuzawa *et al.* 2022, Withey *et al.* 2022, Pérez Barón *et al.* 2024). Recently, functional imaging has gained significant interest in the management of patients with PPGL. These include ^123^I MIBG, ^18^F-FDG PET/CT (fludeoxyglucose 18) and ^18^F-DOPA PET/CT (6-[18F]-L-fluoro-L-3, 4-dihydroxyphenylalanine) and ^68^Ga DOTATATE PET/CT scans. Although ^123^I MIBG imaging is useful in the functional assessment of PPGL with high sensitivity (83–100%) and high specificity (98–100%) in detecting pheochromocytoma, its sensitivity has been downgraded in recent comparative studies, especially in metastatic pheochromocytoma and paraganglioma (mPPGL), *SDHx (*succinate dehydrogenate*)* PPGL (particularly *SDHB*), small tumor and mPPGLs and head and neck paragangliomas (HNPGLs) (Timmers *et al.* 2024). This is because, more aggressive tumors (e.g., fast-growing tumors) or metastatic lesions have a lower expression of tumor cell membrane transporter systems (Timmers *et al.* 2024). ^123^I MIBG has largely been replaced by ^68^Ga DOTATATE imaging, which is found to be more sensitive than other tracers in any *SDHx* PPGLs, mPPGLs and sporadic or hereditary HNPGLs with the lesion-based location rate approaching 100%. Despite comparative studies still lacking, it was suggested to be less sensitive for abdominal PPGLs (Gild *et al.* 2018). ^18^F-DOPA PET/CT has a sensitivity approaching 100% and a high specificity of >95% for the detection of PPGLs but it is substantially lower in metastatic tumors (Timmers *et al.* 2024). It has more specificity in detecting PCC, given it relies on low to moderate tracer uptake by healthy adrenal glands compared with other tracers. The main disadvantage of this tracer is that it is not approved or routinely available in most countries (Timmers *et al.* 2024). ^18^F-DOPA PET/CT is less specific for the detection of these tumors as it reflects the glucose uptake and its metabolism by the cells. However, it is useful in differentiating PPGLs from other cancers such as transitional cell carcinoma and lymphoma (Timmers *et al.* 2024). In addition to ^123^I MIBG-avid and ^68^Ga DOTATATE non-avid bladder mass, our patient’s genetic testing for *SDHx* mutation did not reveal any pathogenic variant and she did not have metastatic disease, which may suggest that she might have better prognosis than other patients who carry the pathogenic variant and/or has metastatic disease. Multiple imaging modalities including functional imaging along with what is available is useful in the assessment of these tumors.

The nonspecific nature of these patients’ symptoms results in misdiagnosis leading to the omission of alpha-blockade before surgery. This may put them at risk of developing complications related to surgery. Indeed, in one case series of patients with PUBs, of 20 patients, only two underwent alpha-blockade prior to surgery (Cai *et al.* 2022). Interestingly, this was despite eight of the patients having preoperative diagnoses of PUB. Moreover, in the 2022 systematic review of 194 cases, only 53% of those diagnosed before surgery had alpha-blockade (Li *et al.* 2022). The evidence for alpha-blockade has been debated (Groeben *et al.* 2017, 2020, Cai *et al.* 2022, Van Den Heede *et al.* 2022); however, given the potential risks, it is advisable that any patients presenting with nonspecific symptoms and a bladder tumor are discussed within a multidisciplinary meeting and that decisions for preoperative preparation and perioperative management are made jointly. The most recent guidance from the Endocrine Society advocates for preoperative alpha-blockade (Lenders *et al.* 2014). Perhaps most importantly, the correct diagnosis should be sought early to optimize management with expert consultation; as such, when there is any suspicion of PUB, plasma or urinary metanephrines must be measured before any surgical intervention. The consequences of misdiagnoses is well illustrated in the case from Jordan (Alkhatatbeh *et al.* 2020) where a patient had to endure three trans-urethral surgical interventions before the completion of treatment (see Table 2). Biochemical workup was only done after histology from the second operation’s sample, despite a hypertensive crisis during the first operation.

Surgical removal of the tumor is the mainstay of treatment in PUBs; however, it requires careful planning via MDTs for various reasons as discussed above. The diagnosis of PUBs can be difficult because of the overlap of symptoms and imaging characteristics with other urinary conditions such as urothelial carcinoma (Male *et al.* 2019, Zare *et al.* 2024). If patients are not diagnosed and managed with alpha-blockade before surgery, there is a significant risk of intraoperative catecholamine crisis and postoperative complications (Lenders *et al.* 2014, Li *et al.* 2022). A recent multicentric study showed only one-third of patients with PUB were diagnosed before surgical intervention, reflecting the need for better diagnostic strategies to prevent intra-/postoperative complications (Yu *et al.* 2022). Partial cystectomy was successfully performed in our patient without any intraoperative or postoperative complication as she was managed under MDT care, with considerable preoperative planning and adequate alpha-blockage before surgery.

Although data on genetic characteristics in PUB are limited, there are few studies that have examined the characteristics of germline mutations in this cohort (Lawrence *et al.* 2004, Park *et al.* 2017, Srirangalingam *et al.* 2017, Yu *et al.* 2022). Although our patient’s genetic testing results came back as negative, around 40–63% of patients with PUB carry a germline mutation and most commonly the *SDHB* subunit gene*,* which requires them to be followed up lifelong because of the increased risk of developing metastatic disease (Withey *et al.* 2022). The importance of monitoring in such cases with *SDHB* mutations is clearly exemplified in the case from Canada (Malhotra *et al.* 2018). The patient was monitored with serial urinary metanephrines and was found to have a recurrence just three years later (Table 2). In our case, immunohistochemistry for the *SDHB* protein in histology was not carried out, but it can be very useful in settings where genetic testing is not available/or limited because of other factors. If the histology sample shows strong *SDHB* protein staining, then the patient is unlikely to carry the germline mutant *SDHB* gene (Park *et al.* 2017). PUB can also be a part of genetic syndrome such as multiple endocrine neoplasia 1 (MEN-1), Von Hipple–Lindau syndrome (VHL) and Carney–Stratakis syndrome (Shi *et al.* 2022).

In conclusion, this case report highlights the importance of considering PUB as a differential diagnosis in patients presenting with post-micturition symptoms and paroxysmal hypertension. Consideration should be given to preoperative medical management with alpha blockers to reduce the risk of intraoperative catecholamine crisis and favorable clinical outcomes after during and after surgery. A comprehensive approach should be taken for imaging techniques with regards to localization and screening of metastases as no medium has full sensitivity. Furthermore, this case underlines the need for a multidisciplinary approach to such cases, including team input from endocrinology, radiology, nuclear medicine, general surgery, urology, pathology and biochemistry in the evaluation, management and future follow-up of such rare and complex cases.

## Declaration of interest

The authors declare that there is no conflict of interest that could be perceived as prejudicing the impartiality of the work.

## Funding

This work did not receive any specific grant from any funding agency in the public, commercial or not-for-profit sector.

## Patient consent

Written consent from the patient was obtained to use anonymized images and to publish the case.

## Author contributions

KMS, JMP and CBA drafted the initial manuscript, reviewed the literature, gathered data and obtained consent from the patient. MW gathered images and reviewed and edited the manuscript. TTC reviewed and edited the manuscript and supervised the study. All authors reviewed, revised and finalized the manuscript.
